# Beijing’ s paired assistance program: Mitigating vulnerability, fostering development, and promoting equality in its ecological conservation areas

**DOI:** 10.1371/journal.pone.0324817

**Published:** 2025-05-29

**Authors:** Guangcai Xu, Jiahui Zhang, Mingyi Xie, Haixia Du, Mengqi Guo

**Affiliations:** 1 School of Economics and Management, Beijing University of Agriculture, Beijing, China; 2 Beijing Research Centre for Rural Revitalization, Beijing University of Agriculture, Beijing, China; 3 Wuxi Branch of Jiangsu Academy of Agricultural Sciences, Wuxi, China.; Vietnam Maritime University, VIET NAM

## Abstract

Interregional cooperation is an important mechanism for promoting regional equality. The paired assistance policy (PAP) has been broadly used to address regional inequality in China. In Beijing, developed plain districts have been paired, one-to-one, with less developed ecological conservation areas (ECAs) in mountainous regions to promote green development and livelihood improvements. In these pairings, lateral inputs from developed regions such as financial inputs, industrial assistance, human resource training, etc., are provided to the ECAs. To analyse the effect of Beijing PAP implementation, the 2013–2022 vulnerability index from each district in the ECAs was evaluated with the Vulnerability-Scoping-Diagram (VSD) model to reflect the changes in the ecological environment before and after PAP implementation. The Propensity Score Matching Difference-in-Differences model (PSM-DID) was used to explore the impact of the PAP mechanism on the green development and livelihoods of rural residents in ECAs. The results revealed that the vulnerability index of the ECAs decreased from 0.45 in 2013 to less than 0.05 in 2022, with an average annual reduction of 9%, attributing to the strict implementation of environment preservation policies. The PAP mechanism also clearly promoted green economy and livelihoods development in the ECAs. In addition to effectively stimulating development in Beijing ECAs, the PAP mechanism can be referenced by similar metropolitan cities to develop regional collaboration for ecological protection and balanced development.

## 1. Introduction

Sustainable development goals (SDGs) have been strengthened to end poverty, fight inequality and injustice, and address climate change by 2030 [1]. The subnational development inequalities have been a general concern of the international community and seen as regional development traps [2]. Examples of such situations include the North‒South Divide in Britain [3], the advanced North and the less developed South of Italy [4], subnational inequality in Mexico [5], and the uneven coastal-inland development in China [6]. Moreover, rural–urban inequalities and their impacts on sustainability have received increasing attention, and complex relationships and interactions have been explored for building links between rural and urban areas [7].

Many countries have been devoted to alleviating the unbalanced development between regions and formulating corresponding policies to reduce regional disparities [8]. Regional development policies, along with redistribution policies [9], have been formulated by governments to promote balanced development via tax base sharing and social policies [10] to favour less developed regions. Common measures to promote balanced regional development include inclusive urbanization systems that extend infrastructure to less developed regions, the implementation of special development strategies in priority areas, and the promotion of regionally driven self-development in less developed regions. However, assistance from external regions also plays a positive role in increasing interregional economic or market access and encouraging the sharing of technical expertise and knowledge [11] through partnerships such as town pairing mechanisms [12] and Sister Cities [13].

China has great regional differences and has been undergoing an unbalanced development. The cooperation between developed and less developed provinces has been encouraged as one of the important policy tools to reduce regional disparities [14]. Since 1979, China has institutionalized the provincial pairing mechanism [15]. In the 21st century, this mechanism has been reinforced [16]. The eastern provinces have extended substantial assistance to the less developed provinces. This is conducted within the framework of the Paired Assistance Policy (PAP).

The PAP mechanism performs well in addressing poverty alleviation [17], natural disaster relief [18], environment management [19]. The PAP mechanism has mainly been implemented at the provincial level [20], while its applications at the county and city level are increasing [21]. Regional PAP mechanisms have been established within Sichuan Province, the Yangtze River Delta, and the Pearl River Delt respectively, with internally developed cities pairing up to support less developed ones [22]. Since 2018, Beijing Municipality has established PAP at the county level, where the counties in plain areas with stronger economies are paired with the poorer counties in Ecological Conservation Areas (ECAs), with the former providing lateral financial support, industrial project cooperation and employment support to the latter.

The PAPs have been successful in reducing regional development disparities [16]. The main PAP measures include the provision of lateral transfer payments, support for industrial development, and the promotion of the labour employment of underdeveloped regions. Most research has focused on the provincial level, including the role of the government in implementing PAPs, the determination of transfer payments [23], and the content of cooperation [24]; In recent decades, there has been a gradual increase in the prevalence of PAPs at the county level, which have not yet attracted sufficient academic attention. However, these programs have directly affected the regional ecology and the inhabitants’ livelihoods at the micro-scale. Most of the counties receiving PAP assistance are ecologically fragile and economically backward, and PAP focuses mainly on economic and social impacts, but the ecological impacts it produces have not been adequately understood, which is not conducive to a comprehensive assessment of the combined effects of PAPs.

The purpose of this study is to analyse the impact of PAP implementation on the assisted areas at county level, and to provide an important basis for decision-making at the higher levels of government. To this end, the PAP programme in Beijing ECAs is taken as a case study, and the main focus is to analyse the ecological impacts of PAPs on assisted areas. Following a systematic review of the implementation process, the ecological impacts of the PAP on ECAs from 2013 to 2022 were assessed using the Vulnerability Scoping Diagram (VSD) framework. A comparison before and after the implementation of the PAP was carried out to differentiate between the level of development of the green economy and the level of sustainability of local livelihoods in ECAs.

The contributions of this study are as follows: First, this study introduces the framework of vulnerability analysis to the field of PAP and evaluates for the first time the impact of the PAP mechanism at the county level on the sustainable occurrence of ecological economy in the assisted areas. Second, the time-varying vulnerability analysis highlights the important role of continuous PAP-based inputs in the development of ecological reserves. Third, this paper establishes a vulnerability assessment system based on existing studies, which can provide a useful reference for future research. The results of this study are expected to be an important reference for the implementation of ecologically oriented and balanced development in metropolitan areas.

## 2. Literature review

### 2.1. The impact of the paired assistance policy

The paired assistance policy represents a governance mechanism for the lateral transfer of resources and cross-border cooperation. The effects of the implementation of PAP have recently been evaluated from a variety of fields and perspectives.

The PAP has been contributing to the social-economic development of the recipient regions. As demonstrated by Li et al., the implementation of the PAP has promoted economic growth in Tibet and Xinjiang through the enhancement of infrastructure and public services [25]. Zhou et al. argued that PAP mechanism is political mobilization rather than institutionalized and the non-equilibrium of interests of both sides, and should be promoted to collaborative development, changing the political mobilization to the systematic incentives to meet the profitable need for both sides [26]. Currently, an increasing number of PAPs conducted in the Yangtze River Delta [27] and the Pearl River Delta [28] collaborate to ensure the mutual benefit while achieving political goals by engaging in interlocal agreements on environmental protection, tourism, immigrant labour, trade, and other matters. Wang believes that the process of PAP is accompanied by an extension of power from one city to another and favouring to fulfil the helping party’s vision of breaking through the limitations of spatial scale [29].

The PAP in the field of great projects mainly focuses on the construction of expansive water conservancy projects, with a particular emphasis on the issues of resettlement and ecological compensation. A case study on the Three Gorges Project revealed that PAP had a considerable positive impact on the economic and social development of the reservoir area by financing the reconstruction of schools, hospitals, and other basic infrastructure [30]. The South-to-North Water Transfer Project represents a case study that focuses on the game process regarding lateral payments based on the PAP framework [31]. In order to ensure the fairness and legitimacy of the lateral ecological compensation standards within the PAP framework, it is essential to consider not only the opportunity cost but also the impact of changes in water quality and the value of water resources [32]. The development of a robust mechanism for regular adjustment of these standards is crucial to ensure the long-term sustainability [33].

Regarding the ecological and environmental effects of the PAP mechanism. The environmental outcomes in the assisted regions show mixed performance. From a positive perspective, the PAP mechanism supported sustainable agricultural development mainly through the fine management of agricultural land resources, and a 1% increase in pairing assistance funds is associated with an increase of about 0.5% in the region’s agricultural sustainability index [34]. However, unexpected negative impacts are also found in the process of PAP. Yu found that while the PAP was intended to curb carbon emissions in the assisted border regions, it inadvertently exacerbated carbon emissions in these regions. This effect was realised by breaking market segmentation and forcing high-carbon firms to exit [35]. So, the environmental effect of PAP mechanism should be well evaluated according to the background.

The effectiveness of PAP policy implementation is influenced by many factors. The number of lateral transfers, the main uses of the inputs, cooperation mechanisms between regions, etc. Li posited that environmental conditions (institutional proximity, geographic proximity, organisational proximity) and cooperative arrangements collectively influence the impact of PAP [27]. A comparative analysis of regional PAP practices has demonstrated that the proximity of the place of assistance to the place of benefit is a significant factor influencing the efficacy of PAP [29].

### 2.2. Vulnerability in policy influences evaluation

The study of vulnerability has a long history, and the IPCC has clarified the relevant concepts of vulnerability in its reports [36]. The study of vulnerability involves multiple aspects of system alteration, sensitivity, potential impacts and adaptation. It covers a number of interrelated areas such as climate change, ecosystem response, and socio-economic development [37]. Vulnerability originated from a focus on socio-ecological systems [38]. It was subsequently introduced into natural domains such as climate change and natural disasters. This led to the development of an integrative research framework that encompasses natural-social and their complex systems. Metzger et al. studied the vulnerability of terrestrial ecosystem services to land use change in Europe [39]. Polsky et al. constructed the Vulnerability Scoping Diagram (VSD), which was compatible with global change [40].

The VSD model consists of three components: Exposure (E), Sensitivity (S) and Adaptive Capacity (AC). Exposure refers to the extent to which a system is affected by external disturbances, including natural factors and anthropogenic activities. The magnitude of exposure depends on the extent of the influence of the stimulus and the spatial location of the system. Sensitivity refers to the degree to which a system responds to external changes and depends on the natural or social features of the system. High sensitivity means more vulnerable to external shocks. Adaptive capacity refers to the system ability to mitigate damage and recover from external pressure, including the available capitals, coping strategies, technical and management systems. The system with high adaptive capacity can better cope with external pressures and reduce vulnerability.

The VSD framework has been widely employed in vulnerability evaluation in the field of climate change, land use, natural disasters, coastal zone management, regional development, heritage conservation [41,42]. And the elements of geospatial mapping, participatory assessment [43], risk evaluation, adaptive management, etc. have been integrated into the framework. However, the relationship between E, S and AC is intricate [44]. Varieties of quantitative methods are used to ascertain the relationships in VSD framework, thereby facilitating assessments and enabling cross-sectional comparisons between systems and time-series analyses of the evolution of system vulnerability [45]. The evaluation of system vulnerability, whether quantitative or qualitative, depends upon the construction of an index system.

Overall, PAPs have played a significant role in promoting balanced regional development in China. Existing studies have focused on the provincial level, mainly through pairing cooperation between developed and less-developed provinces, and the provision of financial, technological, and human resources support to less-developed provinces. The application of the PAP mechanism at the county level has gradually increased, but its effects have not yet been widely and deeply studied, and its comprehensive effects and mechanisms of action have not yet been fully revealed. The methodology of vulnerability assessment plays an important role in studying the ecological effects of regional cooperative development policies. In this study, we therefore select the PAP mechanism at the county level in Beijing and introduce the vulnerability assessment methodology to analyse in depth the impacts brought by the implementation of PAP on the ecological environment of the beneficiary areas and to evaluate its sustainability. We also aim to provide a reference for the promotion of balanced development through regional collaboration in similar metropolitan areas.

## 3. Materials and methods

### 3.1. Study area

Beijing is located on the northern periphery of the North China Plain, between 115^o^25’–117 ^o^ 30’ E, and 39 ^o^ 28’–41 ^o^ 25’ N. Beijing has a land area of 16,410 km^2^. Beijing Ecological Conservation Areas (ECAs) is in the northeast, north and west of Beijing, and is a geographical unit consisting of mountainous areas, alluvial plains and basins between the mountains, accounting for 68% of the Beijing area.

The ECAs is an ecological functional area. However, it is a relatively backward area in terms of economic and social development. This has a detrimental effect on the overall sustainable development of Beijing. The implementation of the PAP has been identified as a key factor in promoting the balanced development of ecological protection and economic and social development at the regional level. The policies and mechanisms employed by the PAP are typical and have the potential to serve as a valuable reference point for similar areas. This selection of the ECA for the present study is primarily based on the recognition of the PAP as a significant initiative in promoting urban sustainability.

**Fig 1:**
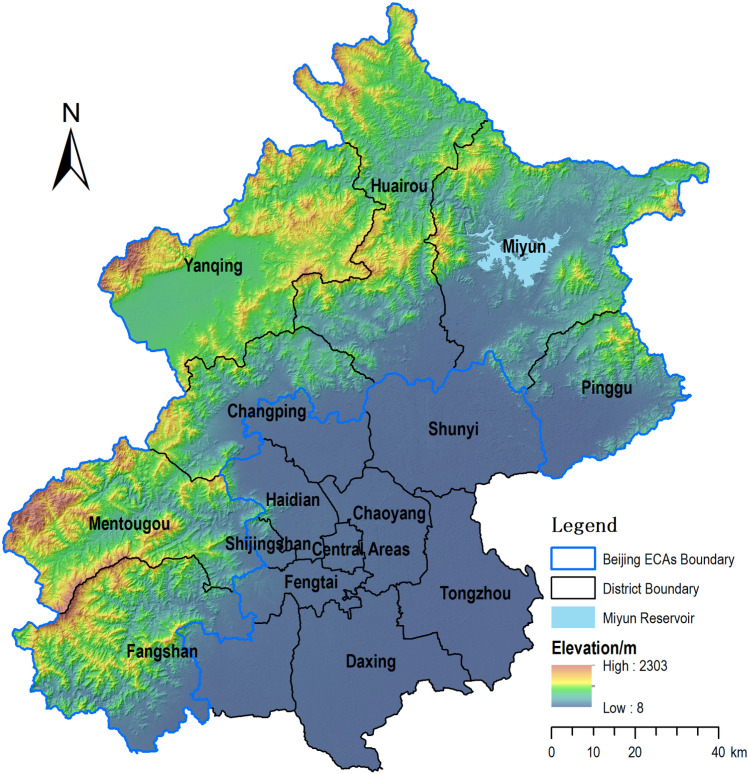
Location of the study area (Software: ArcGIS 10.8.3/www.esri.com/; the review number of the base map: GS [2019] 3333).

The Beijing ECAs (Fig 1) include five full counties (Mentougou District, Pinggu District, Huairou District, Miyun District, and Yanqing District) and the mountainous parts of two counties (Changping District and Fangshan District). *The Master Plan of Beijing (2016–2035)* clearly defined the ECAs as ecological shelter and water source conservation areas of Beijing. Accordingly, the areas are restricted to developing ecological friendly industries, and committed to continuously improve residents’ livelihood by upgrading the infrastructure and public services.

The Beijing ECAs contain approximately 2.62 million residents, 11.98% of the Beijing population, with a density of approximately 22 people/km² in 2022. The region has a semi-humid continental monsoon climate, with hot, rainy summers and cold, dry winters. The terrain declines from both north to south and west to east, and the highest point is the Dongling Peak, which is 2303 m above sea level. The vegetation type is mainly temperate deciduous forest. Approximately 80% of the city’s forest resources, 60% of its water resources and 65% of its wetlands are in ECAs. Beijing ECAs, located upwind of the city and in the upper reaches of the river basin, have historically demonstrated a strong commitment to ecological protection and environmental construction. This has resulted in significant contributions to Beijing’s urban development and supported the prosperity of the plain area of Beijing. However, the remoteness of these areas, their unfavourable locations, and especially industrial limitations have caused them to lag plain areas in economic-social development and residents’ livelihood. In 2022, GDP per capita in the ECA region was 28.5% of that of the plains, and disposable income per capita was 62.3% of that in the plains.

The Beijing Municipal Government has strengthened its financial support for ECAs through vertical transfer payments. However, this is regarded as inadequate. Consequently, the plain areas, which benefit from ecological and environmental protection, were encouraged to provide direct support to ECAs, including direct monetary payments, ecological protection support, and talent training. Such compensation mechanisms are also considered as lateral ecological compensation.

To strictly implement the responsibility of ecological conservation and green development, the Beijing municipal government issued the “*Implementation Measures on Promoting Ecological Protection and Green Development of Ecological Conservation Areas (2019-2022)*” in 2018, which confirmed that ecological conservation was the top priority of ECAs. Seven pairs of partnerships between the ECA counties and the plain counties were established. These include the following partnerships: Dongcheng District and Huairou District, Xicheng District and Mentougou District, Chaoyang District and Miyun District, Haidian District and Yanqing District, Fengtai District and Fangshan District, Beijing Economic and Technological Development Zone and Pinggu District, Shunyi District and Changping District.

The PAP mechanism is innovative for establishing a cross-regional lateral transfer payment system in which the plain district provides financial funds of 600 million CNY (83.58 million USD equally on July 17, 2024) per year directly to ECAs. Districts that are entirely within ECAs receive an annual transfer of $100 million CNY, while districts partially located within ECAs receive an annual transfer amount of $50 million CNY. Furthermore, the plain district provides assistance and guidance in the establishment of green industry projects, while also enhancing the capacity of public services and other forms of support for the economic and social development of ECAs. The PAP mechanism promotes the cooperation and win‒win development of each paired area, gradually evolving into a novel approach to regional coordinated development.

In 2021, “*The Regulations on Ecological Protection and Green Development of Beijing Ecological Conservation Areas”* legalized the above partnership for joint protection of the ecology and environment in the ECAs, which came into force. To ensure the comprehensive implementation of the Regulations and stimulate development vitality in ECAs, *“The Implementation Plan on High-Quality Promotion of Ecological Protection and Green Development of Ecological Conservation Areas in the New Era (2023-2027)”* has been implemented since 2023, when the first round of PAP collaboration expired in 2022.

### 3.2 Regional ecological vulnerability analysis method

#### 3.2.1. Selection of vulnerability indicators.

To measure the influence of PAPs on ECAs, the VSD model was utilized to analyse the dynamic evolution of vulnerability in each district from 2013 to 2022. The construction of the indicators adheres to the principles of suitability, representativeness, and scientific rigor. The selected indicators demonstrate a robust correlation with ECAs referring to previous studies [46], thereby providing a precise and accurate reflection of the local ecological conditions. 22 indicators of three categories, namely, exposure, sensitivity and adaptive capacity, were selected to construct the evaluation system and were divided into 10 subgroups (Table 1).

**Table 1:** Indicator system of the vulnerability assessment of Beijing ECAs.

Subindices	Factor Label	Variables	Reference	Symbol	Unit	Weight	Sign.
Exposure (E)	Population and Urbanization	Population density	[46,49]	x_1_	/km^2^	0.099	(+)
		Urbanization rate	[50]	x_2_	%	0.034	(+)
	Economic Growth	GDP growth rate	[46,51]	x_3_	%	0.006	(-)
		% primary sector value added in GDP	[52]	x_4_	%	0.057	(+)
		% secondary sector value added in GDP		x_5_	%	0.030	(+)
	Meteorological factors	Average annual temperature	[45,53]	x_6_	^o^C	0.051	(-)
		Average annual precipitation		x_7_	mm	0.007	(-)
Sensitivity (S)	Population factor	Natural population growth rate	[54]	x_8_	%	0.021	(+)
	Residents’ livelihood	Per capita income of residents	[55]	x_9_	CNY	0.016	(-)
		Per capita consumption of residents	[56]	x_10_	CNY	0.015	(-)
	Regional Finance	Balance of deposits in local currency	[57,58]	x_11_	10^4^ CNY	0.007	(-)
		Balance of loans in local currency		x_12_	10^4^ CNY	0.006	(-)
	Environmental factor	Total energy consumption (standard coal)	[59]	x_13_	10^4^ t	0.236	(+)
		Average concentration of respirable particulate matter (PM10)		x_14_	μg/m^3^	0.038	(+)
		Volume of sewage discharged	[60]	x_15_	10^4^ m^3^	0.083	(+)
Adaptive Capacity (AC)	Social services	Number of health facilities	[44,45]	x_16_	individual	0.055	(+)
		Number of students in primary and secondary schools		x_17_	10^4^ people	0.069	(+)
	Economic ability	Fiscal expenditure	[45,61]	x_18_	10^4^ CNY	0.041	(+)
		% of the tertiary industry in GDP	[62]	x_19_	%	0.020	(+)
	Environmental protection measures	Decline rate of energy consumption per 10^4^ CNY GDP	[63]	x_20_	%	0.010	(+)
		Sewage treatment capacity	[64]	x_21_	10^4^ m^3^	0.077	(+)
		% Vegetation coverage	[65]	x_22_	%	0.024	(+)

Note: “+” means that the indicator is positive, and “-” means that the indicator is negative.

Exposure is closely related to the natural and social environments in which the system operates. It is important to note that both natural and social risks serve as significant sources of vulnerability [47]. The study starts from the natural and social context of the Beijing ECAs and selects climatic factors (average annual temperature, average annual precipitation), economic growth (GDP growth rate, share of value added of primary sector and secondary sector in GDP), population and urbanization (population density, urbanization rate). Among them, economic growth, population and urbanization indicators, although not external factors, can be used to indicate the overall policy environment to which the local area is exposed.

Sensitivity mainly reflects the properties of the system. These intrinsic factors are susceptible to change by external shocks, which in turn lead to change of the system. Sensitivity indicators include population factor (natural population growth rate), residents’ livelihoods (per capita income and expenditures), regional finance (local deposits and loans), and environmental factors (energy consumption, PM10, sewage discharges). These indicators are susceptible to alteration when the system is affected by shocks from the external environment. For instance, energy consumption is sensitive to climate change and residents’ livelihoods are highly sensitive to changes in the regional economy.

Adaptive capacity is derived from the resources available to the system and its ability to cope with external shocks [48]. Based on the data availability in Beijing ECAs, the indicators of social service supply (number of health facilities, number of students in primary and secondary schools), economic ability (fiscal expenditure, service sector share of GDP), and environmental protection measures (decline rate of energy consumption, sewage treatment capacity, and vegetation coverage) were selected. These indicators are employed in conjunction to respond to the region’s ability to cope with natural and economic risk and shocks.

#### 3.2.2. Ecological vulnerability attributes normalization.

The data were standardized first, and the indicators in the positive and negative directions were standardized according to formulas (1) and (2), respectively. The larger the positive indicators, the higher the vulnerability, and the greater the negative indicators, the smaller the vulnerability.


$$Positive\;indicator,\;X_{ij}^{\prime }=(\frac{X_{ij}-min\{X_{j}\})}{(\max\left\{X_{j}\right\}-min\{X_{j}\}})$$ (1)

$$\;Negative\;indicator,\;X_{ij}^{\prime }=(\max\left\{X_{j}\right\}-\frac{X_{ij})}{(max\left\{X_{j}\right\}-\min\left\{X_{j}\right\}})$$ (2)

where $X_{ij}$ and $X_{ij}^{\prime }$ represent the actual value and the normalized value (0–1) of the indicators in the *j*^th^ column of the *i*^th^ line, respectively, and Max {$X_{j}$} and min {$X_{j}$} represent the maximum value and the minimum value of the *j*^th^ indicators, respectively. The weights (*w*_*j*_) of each variable (*j*) were determined via entropy analysis.

#### 3.2.3. Weights determination.

The entropy method was employed to determine the weights of each indicator [66]. The entropy weight method offers the advantage of automatically adjusting the weights of the criteria in accordance with the uncertainty of the data. It does not need to rely on subjective judgment or expert experience as the AHP method, and it does not require dimensionality reduction and eliminates cumbersome calculations.

#### 3.2.4. Vulnerability index.

The vulnerability index for each district was formulated via the model shown in eq. (3) and eq. (4).


V=E+S−AC (3)

$$\begin{array}{c} E=\sum_{i}^{m}{X^{\prime }}_{ij}w_{j},\;S=\;\sum_{i}^{n}{X^{\prime }}_{ij}w_{j},\;AC=\sum_{i}^{k}{X^{\prime }}_{ij}w_{j}. \end{array}$$ (4)

where *V*, *E*, *S* and *AC* represent the vulnerability index, exposure index, sensitivity index and adaptive capacity index, respectively, and where *i* represents the order of the factor variables.

### 3.3. PAP effect analysis

The implementation of the PAP mechanism in 2018 was treated as a milestone to improve the coordinated development of ECAs, with the introduction of additional capital and other factors of production, the advancement of a green economy, the improvement of local infrastructure, and the enhancement of ecological construction.

To compare the impact of the policy implementation on ECAs, data from several years before and after the implementation date were selected. The concept of the ECA was officially formalized in 2012. The management system for ecological conservation areas in Beijing commenced its gradual establishment and implementation since 2013. Thus, the period 2013–2022 was selected for the analysis of time series. The study utilised a before-and-after approach, examining the impact of the policy implementation on ecological vulnerability.

The implementation of the PAP mechanism has the effect of incentivising the transformation of ecological advantages into economic improvements in ECAs. Therefore, Hypothesis 1 (H1) is proposed: the implementation of the PAP mechanism can promote the development level of the green economy in ECAs.

The initial phase of the PAP mechanism was implemented from 2019–2022. A cross-regional lateral transfer payment system was established and implemented. The wealthier plain district promoted the economic and social development of the paired ECA district by providing financial funds directly or by introducing and establishing green industry projects, increasing the capacity of public services, providing vocational training support, etc. These measures were intended to improve local livelihoods. Therefore, Hypothesis 2 (H2) is proposed: the sustainable level of people’s livelihood in ECAs can be obviously improved by PAP implementation.

#### 3.3.1. Effect assessment model.

Based on assumptions H1 and H2, the green economy development (*GED*) and the livelihood (*LVD*) was selected as the dependent variables to reflect the impacts of the PAP mechanism on of ECAs.


$${GED}_{it}=\beta_{0}+\beta_{1}{Mechanism}_{it}+\beta_{2}{Control}_{it}+\lambda_{i}+\mu_{i}+\varepsilon_{i}$$ (5)

where ${HGED}_{it}$ represents the green economic development level of the i^th^ district in the t^th^ year. As the core explanatory variable, ${Mechanism}_{it}$ represents the implementation of the PAP mechanism of the i^th^ district in the t^th^ year. If the i^th^ district implements the PAP mechanism in a certain year, it is 1; otherwise, it is 0. ${Control}_{it}\;$is a control variable that includes population density, industrial structure, financial expenditure, education and medical care facilities, etc. $\lambda_{i}$ is the individual fixed effect. $\mu_{i}$ is the time fixed effect. $\varepsilon_{i}$ is a random disturbance term.

The influence of ecological conservation-related factors on residents’ livelihoods in ECAs can be alleviated, and the local livelihoods can be improved. Therefore, the livelihood factor was included as a new dependent variable.


$${LVD}_{it}=\beta_{0}+\beta_{1}{Mechanism}_{it}+\beta_{2}{Control}_{it}+\lambda_{i}+\mu_{i}+\varepsilon_{i}$$ (6)

where ${LVD}_{it}$ represents the factor of residents’ livelihood level in the i^th^ district in the t^th^ year.

The DID modelling is a commonly used non-experimental method for policy evaluation. To mitigate the potential impact of the bias due to sample selection and heterogeneity bias caused by the unobserved variables, the Propensity Score Matching method (PSM-DID) was employed to estimate the impact of PAP policy on ecological vulnerability by examining the level of green development and the residents’ livelihoods in ECAs (treatment group) and neighbouring areas (control group).

#### 3.3.2. Variable description.

(1) Dependent variables

The green economic development factor (*GED*) and residents’ livelihoods (*LVD*) were taken as dependent variables. *GED* was constructed with five indicators, including gross regional product (GDP), local currency savings, local currency loans, the sewage treatment rate, and the green coverage rate. The entropy method and the weighted average method were combined to calculate *GED. LVD* was obtained by a weighted average of residents’ income and expenditures.

(2) Explanatory variables

The PAP mechanism (*Mechanism*) was taken as the core explanatory variable. *Mechanism* was obtained by multiplying the ECA districts with the mechanism before and after implementation. The values of *Mechanism* before and after PAP implementation were set to 0 and 1, respectively, for ECA districts and were set to 0 for non-ECA districts.

(3) Control variables

The control variables were selected mainly from three aspects: industrial structure, infrastructure and public facilities. These variables included the share of the secondary sector in regional GDP (*SEG_*), the share of tertiary industry to GDP (*TEG_*), the student number in primary and secondary schools (*Student*) and the number of regional medical facilities (*Medi_*). The indicators of population density (*Density*) and financial revenue and expenditure (*FRE_*) were also introduced in the analysis.

The 7 ECA districts were considered the treatment group, and the 3 neighbouring districts (Tongzhou, Daxing, and Shunyi) in the Beijing Plain area were considered the control group. To facilitate the comparison, another 26 districts from neighbouring Hebei Province and Tianjin city were also included in the control group. The data of all 36 districts in this period were acquired and used to analyse the PAP effect before and after its implementation. All the variables are described in Table 2.

**Table 2:** Descriptive statistics of the variables.

Variable type	Variable name	Express	Mean	Standard deviation
Dependent variable	Green economy development factor	*GED*	717.52	585.95
	People’ s livelihood level	*LVD*	6.88	3.80
Core explanatory variable	Ecological compensation mechanism	*Mechanism*	0.08	0.27
Control variable	Budgetary revenues and expenditures	*FRE_*	245.21	243.63
	Proportion of output value of secondary production to GDP	*SEG_*	0.37	0.16
	Proportion of output value of tertiary industry to GDP	*TEG_*	0.59	0.18
	Population density	*Density*	4725.43	9737.26
	Number of students in primary and secondary schools	*Student*	32.95	47.73
	Number of medical institutions	*Medi_*	2611.30	3576.13

### 3.4. Data sources

The data of 22 variables used in the vulnerability analysis were obtained from the *Beijing Regional Statistical Yearbook (2014--2023)*, statistical year books, and official statistical bulletins of the related 7 ECA districts. With respect to the effect analysis of PAPs, the variable data of the control group were obtained from the *Beijing Regional Statistical Yearbook, Beijing Statistical Yearbook*, *Tianjin Statistical Yearbook* and *Hebei Statistical Yearbook*. The environmental data come from the eco-environmental statistical bulletins of Beijing, Tianjin and Hebei Provinces. The interpolation method was employed to fill in the missing values of several variables for specific years.

## 4 Results

### 4.1. Vulnerability analysis

#### 4.1.1. Spatial–temporal variation in the vulnerability of ECAs.

**Fig 2:**
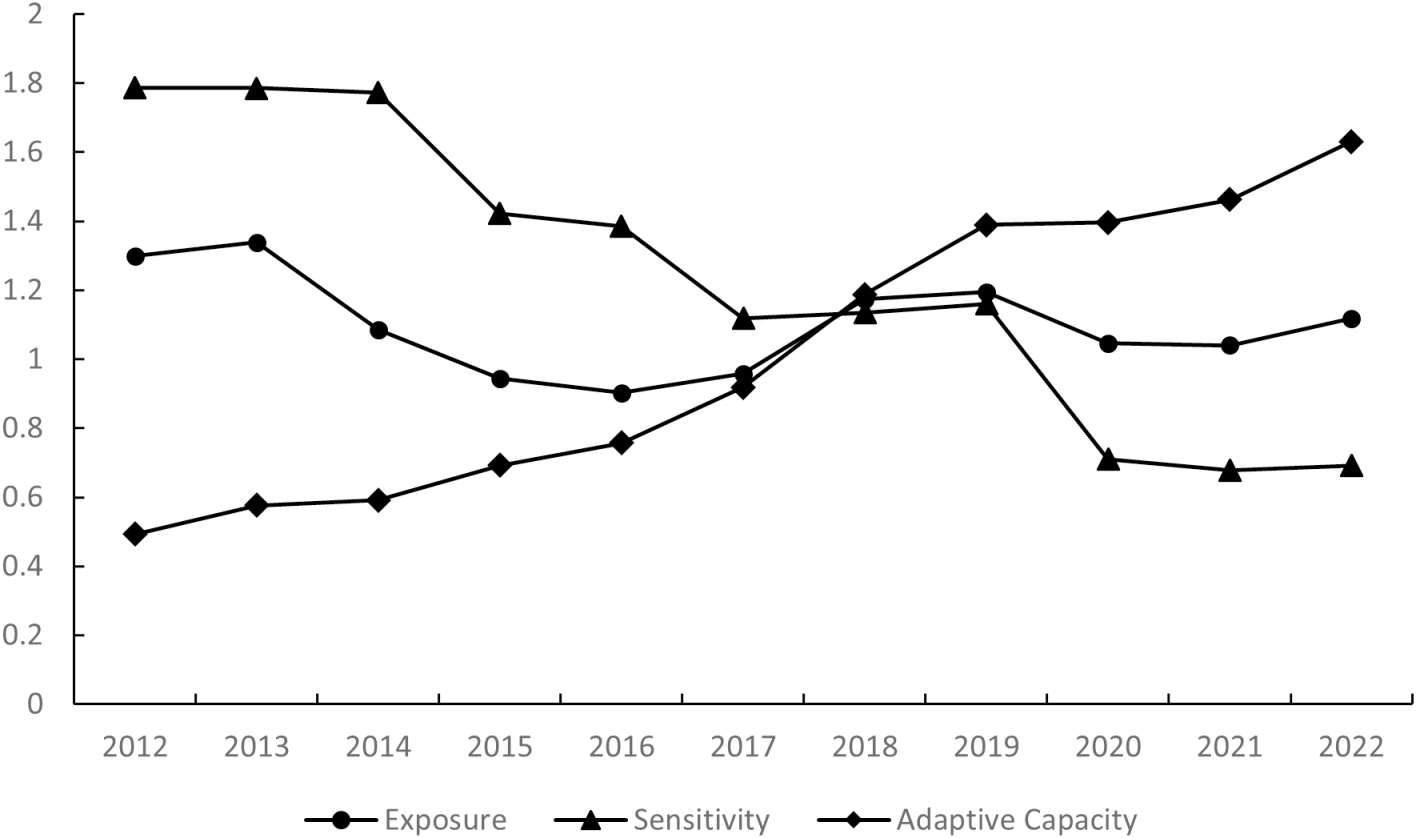
Interannual vulnerability level of each district from 2013-2022.

The vulnerability indices of all ECA districts showed a decreasing trend from 2013 to 2022 (Fig 2), indicating a notable enhancement in the level of ecological sustainability. Despite the notable decline in the level of vulnerability following the implementation of the PAP mechanism in 2019, time series analysis revealed that the downward trend would have continued even without the PAP. In addition, the vulnerability indices of all districts except Changping exhibited a marked decline, reaching below 0.1 in 2020. The vulnerability of Beijing ECAs decreased to a relatively low level, reflecting a regional elevation of sustainability. The decline in the vulnerability index is attributed to Beijing’s strict ecological protection policies in ecological conservation zones, which delineate red lines for ecological protection, limit the scope of urban expansion, and restrict the development of industries such as manufacturing and water-intensive agriculture, as well as encourage the development of eco-agriculture, tourism, and other environmentally friendly industries.

The linear fittings were performed using data from the past 10 years. Segmental linear fittings were performed with the data for 2013–2018 and 2019–2022, and the slopes of the fitted curves provided an indication of the trend variation in the vulnerability index. As shown in Table 3, four of the five complete regions presented significant increases in the slope of vulnerability reduction after the implementation of PAPs, which were greater than those before implementation. The other two regions had their data excluded because only part of the region belongs to the ECAs.

**Table 3:** Slopes of simple linear fitting curves for different ECA districts.

Durations	Pinggu	Yanqing	Mentougou	Miyun	Huairou
Before PAP (2013–2018)	-0.051	-0.025	-0.049	-0.028	-0.038
After PAP (2019–2022)	-0.055	-0.066	-0.020	-0.029	-0.043
Overall duration (2013–2022)	-0.040	-0.025	-0.039	-0.036	-0.036

From the perspective of the districts, Huairou District had the lowest vulnerability index value (0.009) in 2022, followed by Changping (0.013), Yanqing (0.022), Pinggu (0.025) and Miyun (0.028), while Mentougou (0.044) and Fangshan (0.037) had relatively high values. The vulnerability index of Changping District decreased most obviously among all 7 districts. Its exposure declined from 0.19 to 0.14, the sensitivity decreased from 0.3 to 0.12, and the adaptive capacity increased from 0.06 to 0.25. Since 2013, Changping District recalibrated its industrial structure, adopting more ecologically orientated development, resulting in the substantial enhancements in environmental indicators and improving the regional facilities and improving a vigorous economic development.

#### 4.1.2. Analysis of subindexes of the ECA vulnerability over time.

**Fig 3:**
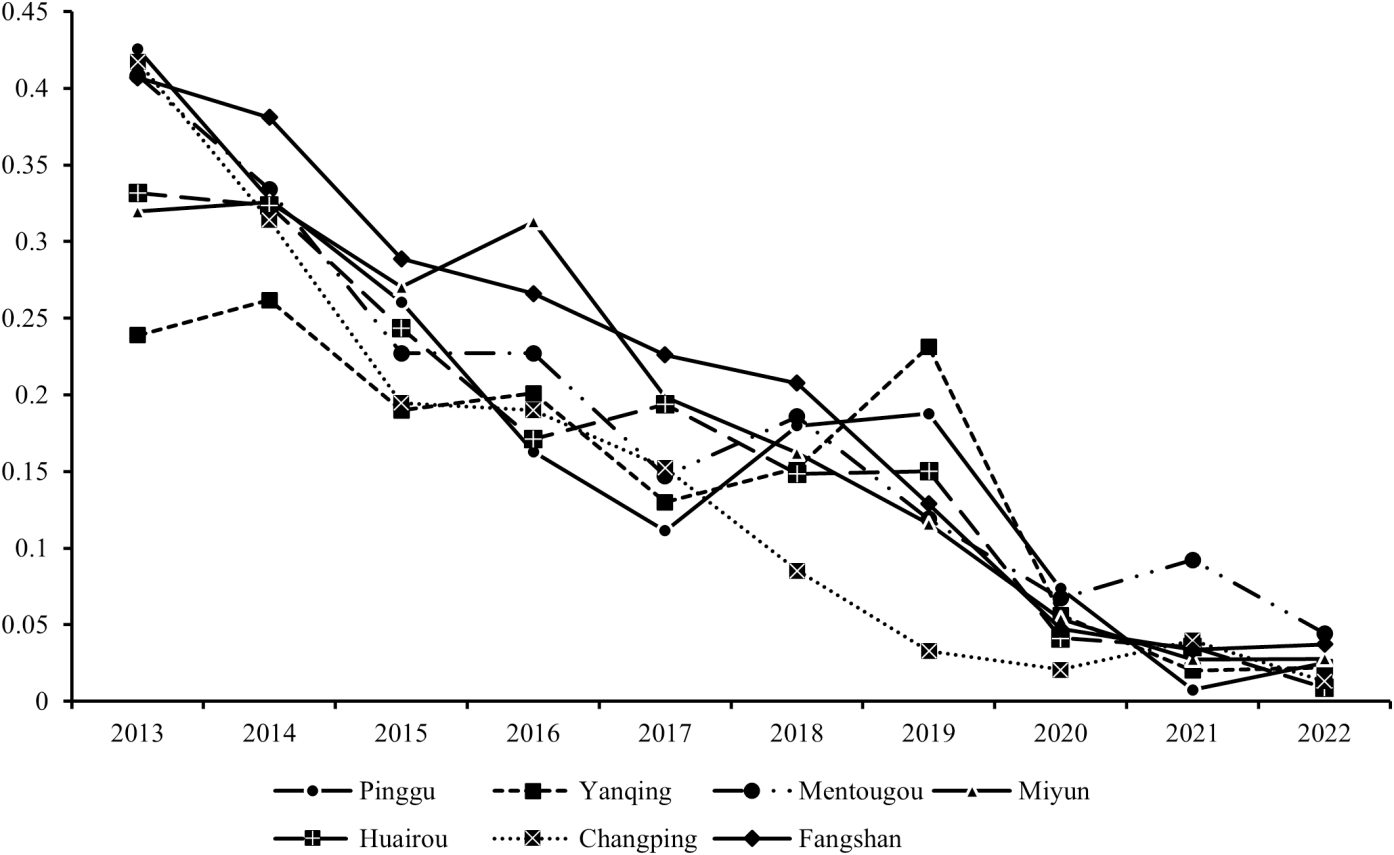
Annual variation in subindexes of vulnerability in Beijing ECAs from 2013-2022.

As shown in Fig 3, the exposure index and sensitivity index of the ECAs before 2018 were higher than the adaptive capacity index, and the sensitivity index was the highest among the three indices. This situation was attributed to strict industrial limitations related to the unfavourable impact of ecological protection policies. After 2018, adaptability, in turn, showed a rising trend and exceeded the other two indices. However, the exposure level demonstrated a fluctuating trend, with a notable decline observed in 2019. The sensitivity level started to decrease significantly (<1) in the second year after PAP implementation. Therefore, the implementation of the PAP mechanism apparently reduced the unfavourable impacts on ECAs by introducing more administrative resources and policy support.

The slopes of the linear fitting curves of exposure, sensitivity and adaptive capacity in Fig 3 were -0.0131, -0.126 and 0.1225, respectively. This reflects that the increase in the adaptive capacity indicator was accompanied by significant overall decreases in the exposure and sensitivity indicators in Beijing ECAs. This trend was also consistent with the changes in the individual ECA districts. This finding also reaffirms that the PAP mechanism and the included policy support had significant positive effects for the reduction of the ecological vulnerability level of the ECAs.

### 4.2. Empirical analysis of PAPs

#### 4.2.1. Balanced test of Propensity score matching.

As shown in Table 4, for the two dependent variables, *GED* and *LVD*, most of the interfering variables in the treated and control groups were significantly different before matching. After matching, the standard errors of all covariates except the medical level and the financial revenue and expenditure were less than 20%, which indicated that the selected covariates and the matching method were reliable. And the results of t-test did not reject the original hypothesis that there was no systematic difference between the treatment and control groups. PSM had a good effect for green economy development factor and livelihood factor.

**Table 4:** Balanced test of Propensity score matching (PSM).

Dependent variables	Variable	Matched	Treated	Control	%bias	t-test	p
*LVD*	*Density*	U	5.176	5.393	-26.7	-1.19	0.000
		M	5.246	5.274	-2.9	-0.22	0.827
	*FRE_*	U	13.280	12.740	46.4	3.22	0.000
		M	13.153	13.216	-6.0	-0.49	0.621
	*SEG_*	U	0.343	0.438	-67.0	-6.94	0.000
		M	0.349	0.348	1.3	0.09	0.929
	*TEG_*	U	0.516	0.392	81.2	8.46	0.000
		M	0.512	0.496	10.8	0.75	0.454
	*Medi_*	U	7.188	6.965	39.3	3.60	0.000
		M	7.357	7.224	23.5	1.77	0.079
	*student*	U	10.169	10.827	-84.1	-1.68	0.000
		M	10.332	10.220	18.6	1.19	0.235
*GED*	*Density*	U	5.176	5.393	-26.7	-1.19	0.000
		M	5.248	5.262	-1.4	-0.10	0.919
	*FRE_*	U	13.280	12.740	46.4	3.22	0.000
		M	13.136	12.920	20.6	1.94	0.053
	*SEG_*	U	0.343	0.438	-67.0	-6.94	0.000
		M	0.350	0.338	5.6	1.03	0.303
	*TEG_*	U	0.516	0.392	81.2	8.46	0.000
		M	0.494	0.502	-8.3	-1.23	0.219
	*Medi_*	U	1431.500	1365.300	7.2	0.62	0.536
		M	1470.600	1411.500	6.4	0.48	0.631
	*student*	U	10.169	10.827	-84.1	-1.68	0.000
		M	10.218	10.115	17.0	1.12	0.262

Notes: U and M were short for unmatched and matched respectively.

#### 4.2.2. Benchmark regression analysis.

The DID model was employed to explore the differences in regional green economy development and residents’ livelihoods before and after the implementation of PAPs. In Table 5, for the development effect of the green economy (*GED*), the PAP (*Mechanism*) had a positive effect at the 5% significance level, demonstrating an incentive effect on the green economy development factor of PAP implementation in ECA districts. After the control variables were introduced, the *Mechanism* coefficient was reduced, and a significant positive effect remained; meanwhile, two variables, *density* and *FRE*_, also had positive effects on *GED*. The PAP was carried out mainly through lateral financial support and green industry development, and the policy improved the economic development by increasing financial income, expenditures, regional GDP and the number of green industries, thus confirming Hypothesis 1.

**Table 5:** Benchmark Regression Results.

Variable	*GED*			*LVD*		
**Fe**	**Fe**	**Re**	**Fe**	**Fe**	**Re**
*Mechanism*	0.037^**^	0.042^**^	0.044^*^	0.189^***^	0.108^***^	0.058
	(0.022)	(0.023)	(0.023)	(0.042)	(0.038)	(0.037)
*Density*		-0.007^*^	-0.005		-0.032	0.120^***^
		(0.021)	(0.018)		(0.035)	(0.021)
*FRE_*		0.020^*^	0.024^**^		0.399^***^	0.368^***^
		(0.011)	(0.011)		(0.019)	(0.017)
*SEG_*		-0.087	-0.072		0.732^***^	0.882^***^
		(0.062)	(0.062)		(0.105)	(0.098)
*TEG_*		-0.024^*^	-0.026		-0.155	0.083
		(0.066)	(0.066)		(0.111)	(0.104)
*Medi_*		-0.015	-0.009		-0.082^***^	-0.044
		(0.014)	(0.014)		(0.024)	(0.048)
*Student*		0.023^*^	0.033^**^		-0.415^***^	-0.403^***^
		(0.014)	(0.014)		(0.022)	(0.021)
*_cons*	8.889^***^	8.630^***^	8.413^***^	9.037^***^	5.838^***^	8.672^***^
	(0.012)	(0.237)	(0.226)	(0.023)	(0.307)	(0.303)
Region	Yes	Yes	Yes	Yes	Yes	Yes
Year	Yes	Yes	Yes	Yes	Yes	Yes
*N*	290	290	290	290	290	290
*R* ^*2*^	0.052	0.053	0.008	0.740	0.789	0.638

Notes:

*p < 0.05,

**p < 0.01,

***p < 0.001. The values in parentheses are standard errors of the estimated values. The same applies below.

The implementation of the PAP mechanism was significantly positively correlated with the livelihoods, and obviously higher than the green economy development. The PAP increased the financial revenue to ECA districts, leading to an increase in the income of residents and in job opportunities via shared employment opportunities with wealthy plain districts. Additionally, the PAP improved the infrastructure of ECAs, which led to the increase of the green industry investment. The implementation of PAPs effectively improved the sustainability of ECAs, which proved Hypothesis 2.

#### 4.2.3. Robustness test.

(1) Parallel trend test

The parallel trend test was employed to test the validity of the DID. The method basically compared the trends of the treatment group and the control group before and after the implementation of PAP. According to Table 6, before PAP implementation, there were mostly positive impacts on *GED* and *LVD*, but none of them were significant. There was no trend difference between the treatment group and the control group before the implementation of the mechanism. The year in which the mechanism was implemented had a significant positive effect on the *GED* and *LVD* variables. The regression coefficients of *GED* and *LVD* were significantly positive after the implementation year and thereafter, indicating that the PAP mechanism obviously increased green economy development and local livelihoods.

**Table 6:** Results of the parallel trend test.

Variable	*GED*		*LVD*	
**(1)**	**(2)**	**(1)**	**(2)**
Pre_6	-0.057	-0.051	-0.222	-0.127
	(0.024)	(0.023)	(0.107)	(0.098)
Pre_5	-0.000	0.005	-0.002	0.049
	(0.008)	(0.007)	(0.038)	(0.039)
Pre_4	0.018*	0.020*	-0.010	0.030
	(0.010)	(0.010)	(0.030)	(0.032)
Pre_3	0.015	0.015	0.005	0.038
	(0.004)	(0.004)	(0.025)	(0.027)
Pre_2	0.003	0.003	0.027	0.050
	(0.007)	(0.006)	(0.017)	(0.018)
Current	0.004	0.002	-0.021*	-0.021*
	(0.006)	(0.006)	(0.012)	(0.013)
Post_1	0.002**	0.001**	0.002**	0.011**
	(0.003)	(0.002)	(0.031)	(0.027)
Post_2	0.006**	0.005**	0.028*	0.003**
	(0.006)	(0.006)	(0.034)	(0.032)
Post_3	0.007*	0.004**	0.067	0.018*
	(0.005)	(0.004)	(0.042)	(0.038)
Control variable	NO	Yes	NO	Yes
District	Yes	Yes	Yes	Yes
Year	Yes	Yes	Yes	Yes
*N*	290	290	290	290
*R* ^*2*^	(0.920)	(0.919)	(0.892)	(0.859)

Notes: Pre_ and Post_ indicated before and after the implementation of the mechanism, respectively, e.g., post_1 means 1 year after implementation. Current represented the year of implementation.

(2) Change in the PAP implementation year

To eliminate the errors caused by different means of identifying the time of policy implementation, the replacement of implementation time was often used for robustness analysis in two ways. The first way was placebo tests, adjusting the time of implementation of the policy by advancing or lagging the policy timing for certain years. The second was to shorten the time window.

First, the placebo test method was used to examine robustness. Table 7 showed that the coefficients of *Mechanism_1* were positive but not significant when the implementation time of the mechanism was advanced by one year, indicating that the first two years of the implementation of the PAP mechanism had no significant effect on the *GED* and *LVD* variables in ECAs.

**Table 7:** Replacement mechanism time.

Variable	*GED*		*LVD*	
**(1)**	**(2)**	**(1)**	**(2)**
*Mechanism _1*	0.040		0.081	
	(0.025)		(0.033)	
*Mechanism 1*		0.043^**^		0.077*
		(0.020)		(0.041)
_cons	8.650^***^	8.616^***^	10.087***	10.145***
	(0.236)	(0.237)	(0.388)	(0.386)
Control variable	Yes	Yes	Yes	Yes
Region	Yes	Yes	Yes	Yes
Year	Yes	Yes	Yes	Yes
*N*	290	290	290	290
*R2*	0.053	0.053	0.802	0.802

Notes: *Mechanism _1* and *Mechanism 1* mean advancing and deferring by one year, respectively.

The coefficients for the *Mechanism_1* were 0.043 and 0.077, respectively. Both were significant, indicating that the implementation of the PAP mechanism for one year could significantly improve the level of *GED* and *LVD* variables in ECAs. This further proves the robustness of the benchmark regression results in the previous section.

Second, to diminish the deviation arising from specific data on the model estimation, the DID regression model with a shortened time window was employed to compare the effects on *GED* and *LVD* variables before and after the PAP implementation. Two-time windows were chosen for analysis, including 3 years before and after implementation (2016–2021), 4 years before and after implementation (2015–2022). Table 8 showed significantly positive coefficients of *GED* and *LVD*, indicating that the effect of the PAP mechanism remains unchanged regardless of the time window chosen. The implementation of the PAP mechanism significantly increased the livelihood and green economic development in the ECAs, thereby reaffirming the accuracy of the hypotheses.

**Table 8:** The estimated results of robustness test based on shortening the time window.

Variable	Before and after 3 years		Before and after 4 years	
***GED***	***LVD***	***GED***	***LVD***
*Mechanism*	0.127*	0.160***	0.141**	0.218***
	(0.074)	(0.021)	(0.064)	(0.025)
*_cons*	8.866***	10.524***	8.880***	10.496***
	(0.017)	(0.049)	(0.015)	(0.056)
Region	Yes	Yes	Yes	Yes
Year	Yes	Yes	Yes	Yes
*N*	202	202	264	264
*R2*	0.029	0.950	0.024	0.915

(3) Changing matching methods

The study employed three matching methods, nearest-neighbour matching, radius matching and kernel density matching, and selected the share of output value of secondary industry in GDP (SEG_), the share of output value of tertiary industry in GDP (TEG_), the number of primary and secondary school students, the number of medical institutions, population density, and the financial revenue and expenditure status (FRE_) as the covariates. Following the matching of the indicators as covariates, regression analyses were conducted to verify the robustness of the baseline regression.

The findings in Table 9 indicated that, apart from the green economy development level, which demonstrated no significance in the DID regression following radius matching, all other matching outcomes showed a significantly positive correlation. This further substantiated the hypothesis that the PAP mechanism had a favourable impact on the enhancement of green economy development and the livelihoods in the ECAs. This outcome served to corroborate both hypotheses H1 and H2. However, the findings also demonstrated that the degree of green economic development in the ECAs was not invariably stable and was considerably associated with the matching outcomes. A certain degree of fluctuation was observed, and its regression coefficients (0.025, 0.058, 0.046) were lower than those of the livelihood (0.148, 0.127, 0.139). This indicated that the stability of the level of green economic development in the ECAs required further enhancement.

**Table 9:** Results after changing the matching method.

	Nearest neighbour matching		Radius matching		Kernel Matching	
*GED*	*LVD*	*GED*	*LVD*	*GED*	*LVD*
*Mechanism*	0.025*	0.148***	0.058	0.127^***^	0.046^*^	0.139^***^
	(0.014)	(0.097)	(0.058)	(0.050)	(0.028)	(0.069)
_cons	9.025***	10.852***	5.784***	7.232***	8.313***	9.403***
	(0.114)	(0.817)	(0.173)	(0.151)	(0.187)	(0.291)
*Control Variable*	Yes	Yes	Yes	Yes	Yes	Yes
*Region*	Yes	Yes	Yes	Yes	Yes	Yes
*Year*	Yes	Yes	Yes	Yes	Yes	Yes
*N*	142	142	290	290	218	218
*R* ^2^	0.094	0.226	0.103	0.652	0.011	0.343

## 5. Discussions

Transregional management issues present a significant challenge. In recent decades, China has enforced multiple regulations regarding ecological protection compensation, which are expected to help solve these issues [67]. The PAP is a very important starting point for ecological compensation. It adheres to a problem-oriented approach and establishes a direct correspondence, characterized by high-level coordination, between ecological beneficiaries and those who protect the environment. This relationship can help resolve difficulties and obstacles in lateral ecological compensation [68].

Beijing, as a megacity, has adopted two approaches to ecological compensation in ECAs. First, the municipal government makes transfer payments to the ecological zones for local ecological protection, infrastructure and public service construction, and farmers’ livelihood improvements. This approach is known as vertical ecological compensation. Second, the PAP mechanism has been implemented under the guidance of the municipal government, with each beneficiary area providing compensation, which is known as lateral ecological compensation [69].

The annual funding of the vertical ecological compensation is 4 billion yuan [70], which is approximately 6.7 times of the amount of lateral ecological compensation. The objective of vertical compensation is to improve the supply of ecological services and improve regional ecological security; the objective of lateral compensation is to solve the problem of reciprocity of rights, responsibilities and benefits between regions, and to promote inter-regional ecological environment co-construction and green development and benefit sharing. This study mainly focused on the changes of ecological effects in the ECAS before and after the implementation of lateral ecological compensation, and further analysis is needed to optimize the superimposed effects of lateral and vertical compensation, as well as the ratio of the direct amount of lateral and vertical compensation in the future. Meanwhile, a new idea of comprehensive compensation mechanism combining vertical and lateral compensation has been brought forward, and the construction of this mechanism needs to further strengthen the role of lateral compensation.

The PAP mechanism establishes a compensation relationship between areas that do not have direct adjacency. The mechanism is coordinated by the municipal government, regulated by a set of laws and regulations, and administered by a specialized municipal department, which regulates the criteria and content of the compensation and monitors the implementation effects. In this process, administrative and market-oriented mechanisms work together [71]. The former is used mainly to determine relationships between regions. The latter is employed primarily by plains areas that enjoy ecological benefits. Local enterprises are guided to utilise market-oriented mechanisms in areas with ecological benefits to invest in ECAs and develop industries suitable for ECAs.

A variety of approaches have been used in the vulnerability assessment process [72], including those based on spatial mapping and indicator weighting. In this process, the equal weighting approach is typically used to evaluate exposure, sensitivity and adaptive capacity [53]. This process may neglect the variability of the various study subjects and the differences in the importance of each factor in the vulnerability system. Therefore, in this study, the entropy value method was adopted to determine the weights of the indicators within the vulnerability evaluation system. This approach represents a further attempt to utilize existing evaluation methods.

The development of a green economy and the enhancement of the residents’ livelihoods are significant for the vulnerability reduction. This study investigated the impact of the PAP mechanism on the green economy development and the livelihoods using PSM-DID method. The findings substantiate the notion that the PAP mechanism has been exerting favourable influences on the vulnerability alleviation. Nevertheless, the PSM-DID analysis was inadequate in its depth, thus necessitating the inclusion of additional mediating variables to more comprehensively assess the PAP mechanism’s impacts. Additionally, the study did not incorporate a heterogeneity analysis due to the limited sample size observed in the ECAs region.

It is anticipated that the PAP Mechanism will gradually mature into a stable framework for cooperation, capable of evolving as needed. The first round of the PAP in Beijing ECAs primarily centred on the institutionalization of financial support and the delineation of cooperation areas. The 100 million RMB/year of funds directly transferred from the plain counties could only cover a small share of the inputs needed of the recipient counties in promoting future ecological construction, green industry development, and livelihood support. To further enhance the collaboration, it is essential for the ECAs counties to introduce more market-oriented enterprises, further revitalizing local resources and participating in the green industries, thereby facilitating industrial upgrading. Regarding social security within ECAs, it is of the utmost importance to reinforce the social security system, augment investment in education, skill training and employment through the PAP mechanism. Furthermore, both parties can leverage their respective strengths to jointly advance the ecological conservation, foster industrial cooperation, and facilitate resource sharing across regions, thereby achieving mutually beneficial advantages.

## 6. Conclusion

The elements presented in the results illustrate the variations in vulnerability and effectiveness of the PAP mechanisms. In the long run, the level of vulnerability has decreased since the establishment of the ECAs. The exposure level tended to be staggered during 2013–2022, whereas the sensitivity generally decreased, reflecting a reduction in exogenous factors that stimulate ecological vulnerability. Furthermore, the adaptive capacity tended to continuously increase, which was attributed to long-lasting ecological conservation and infrastructure construction in recent decades.

A decrease in vulnerability was observed to be associated with the influence of numerous factors and was not notably correlated with the PAP program. Since the establishment of the ECAs, the government has enforced rigorous ecological conservation policies and industrial restrictions to ensure the continuous improvement in local ecological functions. The PAP program, which has been in place since 2019, was only one of many such programs. However, it contributed positively to reducing vulnerability and obviously contributed to the development of a green economy and improvements in the livelihoods of residents in the ECAs. However, its effect on the sustainability of the livelihoods of residents seems to be greater than that on the development of the green economy in ECAs.

PAP, a decades-long practice in China, has yielded remarkable results. It has effectively addressed resource shortages in underdeveloped regions through comprehensive support in funding, talent, and technology, fostering resource aggregation and development. Simultaneously, PAP significantly advances the SDG goals of these regions, narrowing the regional development gap and fostering harmony and solidarity among regions.

The success of this mechanism would have been inconceivable without the coordination of the Beijing Municipal Government. This situation suggests that economically developed cities and regions can facilitate the sustainable development of their own functional ECAs through intraregional collaboration. In this process, it is imperative that high-level governments need to designate sound policies and effective implementation safeguards to ensure the smooth implementation of this mechanism and the attainment of the desired results. This research could provide useful lessons for similar regions, especially large cities and urban agglomerations. It could assist in formulating suitable policies aimed at promoting balanced development between developed and less developed regions (including ecological functional areas) within their boundaries. This could be achieved through mutual collaboration in the following areas: financial subsidies, technology transfers, human resources training, and employment support.

This study provides a solid theoretical foundation for implementing the PAP mechanism in Beijing’s ECAs. However, it’s crucial to acknowledge its limitations, especially concerning regional scope and vulnerability indicator construction. Focusing solely on Beijing’s ECAs might affect the representativeness and generalizability of our findings. Future research should broaden its regional scope to include a variety of ecological systems, enhancing the applicability of findings in diverse contexts. In addition, the selection of ecological vulnerability indicators in our study, while grounded in region-specific contexts and previous research, may not be comprehensive in its scope. Future investigations should broaden research horizons, integrating more variables into analyses to capture the multifaceted nature of ecological vulnerability. Ultimately, future study should concentrate on assessing the long-term ramifications of the PAP mechanism and evaluating the efficacy of various implementation strategies.

## References

[1] UN. Transforming our world: The 2030 agenda for sustainable development. New York. 2015.

[2] Diemer A, Iammarino S, Rodríguez-Pose A, Storper M. The Regional Development Trap in Europe. Economic Geography. 2022;98(5):487–509. doi: 10.1080/00130095.2022.2080655

[3] Rowthorn R. Combined and Uneven Development: Reflections on the North–South Divide. Spatial Economic Analysis. 2010;5(4):363–88. doi: 10.1080/17421772.2010.516445

[4] Daniele V. Two Italies? Genes, intelligence and the Italian North–South economic divide. Intelligence. 2015;49:44–56. doi: 10.1016/j.intell.2014.12.004

[5] Gutierrez JP, Agudelo-Botero M, Garcia-Saiso S, Zepeda-Tena C, Davila-Cervantes CA, Gonzalez-Robledo MC, et al. Advances and challenges on the path toward the SDGs: subnational inequalities in Mexico, 1990-2017. BMJ Glob Health. 2020;5(10):e002382. doi: 10.1136/bmjgh-2020-002382 33122296 PMC7597504

[6] Kanbur R, Wang Y, Zhang X. The great Chinese inequality turnaround. Journal of Comparative Economics. 2021;49(2):467–82. doi: 10.1016/j.jce.2020.10.001

[7] Liu C, Valentine G, Vanderbeck RM, Diprose K, McQuaid K. Rural–urban inequality and the practice of promoting sustainability in contemporary China. GeoJournal. 2018;84(5):1187–98. doi: 10.1007/s10708-018-9915-y

[8] Evans M, Marsh D, Stoker G. Understanding localism. Policy Studies. 2013;34(4):401–7. doi: 10.1080/01442872.2013.822699

[9] OECD. OECD Regional outlook 2023: The longstanding geography of inequalities. Paris: OECD Publishing; 2023. https://www.oecd.org/en/publications/oecd-regional-outlook-2023_92cd40a0-en.html

[10] Novak K. Economic linkages: greater minnesota and the twin cities. J Minn Acad Sci. 1990;55(2):6–12.

[11] Zelinsky W. The Twinning of the World: Sister Cities in Geographic and Historical Perspective. Annals of the Association of American Geographers. 1991;81(1):1–31. doi: 10.1111/j.1467-8306.1991.tb01676.x

[12] Brakman S, Garretsen H, Oumer A. Town Twinning and German City Growth. Regional Studies. 2015;50(8):1420–32. doi: 10.1080/00343404.2015.1023282

[13] Baycan-Levent T, Gülümser Akgün AA, Kundak S. Success Conditions for Urban Networks: Eurocities and Sister Cities. European Planning Studies. 2010;18(8):1187–206. doi: 10.1080/09654311003791259

[14] Lu Z, Deng X. China’s Western Development Strategy: Policies, Effects and Prospects. Munich Personal RePEc Archive. 2011; 35201. https://mpra.ub.uni-muenchen.de/35201//

[15] Song T, Liu W, Liu Z, Wuzhati Y. Policy Mobilities and the China Model: Pairing Aid Policy in Xinjiang. Sustainability. 2019;11(13):3496. doi: 10.3390/su11133496

[16] Lu S, Ma J. East-west pairing assistance and cross-regional investment. J Tongji Univ (Soc Sci Ed). 2024;35(2):114–28.

[17] Wang Q, Tian Z, Zhu S. Paired assistance and poverty alleviation: Experience and evidence from China. PLoS One. 2024;19(2):e0297173. doi: 10.1371/journal.pone.0297173 38363780 PMC10871504

[18] Zhong K, Lu X. Exploring the administrative mechanism of China’s Paired Assistance to Disaster Affected Areas programme. Disasters. 2018;42(3):590–612. doi: 10.1111/disa.12262 29086990

[19] Börner J, Baylis K, Corbera E, Ezzine-de-Blas D, Honey-Rosés J, Persson UM, et al. The Effectiveness of Payments for Environmental Services. World Development. 2017;96:359–74. doi: 10.1016/j.worlddev.2017.03.020

[20] Huang Z. Evaluation on the Effect of Investment in Employment and Poverty Alleviation under the Guidance of Urban Pairing Assistance Policy. AJMSS. 2023;3(3):179–82. doi: 10.54097/ajmss.v3i3.11129

[21] Qiu T, Luo B, Li Y. Economic performance of the pairing-off poverty alleviation between China’ cities. Cities. 2024;152:105231. doi: 10.1016/j.cities.2024.105231

[22] Zeuthen JW. Rescaling China’s rural–urban frontier: Exception as norm in the access to development. China Information. 2020;34(2):208–28. doi: 10.1177/0920203x20920817

[23] Guo M, Xie M, Xu G. Sustainable Livelihood Evaluation and Influencing Factors of Rural Households: A Case Study of Beijing Ecological Conservation Areas. Sustainability. 2023;15(13):10743. doi: 10.3390/su151310743

[24] Gastineau P, Mossay P, Taugourdeau E. Ecological compensation: How much and where?. Ecological Economics. 2021;190:107191. doi: 10.1016/j.ecolecon.2021.107191

[25] Li X, Wang G, Duan P, Huang J. Paired assistance: policy rationale and economic performance as illustrated by Xizang and Xinjiang. China Economist. 2024;19(3):70–88. doi: 10.19602/j.chinaeconomist.2024.05.05

[26] Zhou X, Ma X. From “the counterpart support” to “the collaborative development”: enlightenment of the collaborative governance model. Nanjing J Soc Sci. 2012;9:67–7. doi: 10.15937/j.cnki.issn1001-8263.2012.09.016

[27] Lin B, Wang J. Will the “Pairing Assistance” Policy Trigger the Migration of Polluting Enterprises? An Empirical Study Based on the Yangtze River Delta Region. Sustainability. 2023;15(3):1942. doi: 10.3390/su15031942

[28] Chen B, Suo L, Ma J. A Network Approach to Interprovincial Agreements. State and Local Government Review. 2015;47(3):181–91. doi: 10.1177/0160323x15610384

[29] Wang X, Zhao Y. The production of extended local territory: Topology and the spatial politics of city-region making in China. Urban Stud. 2025;62(5):995–1014. doi: 10.1177/00420980241270959 40151343 PMC11937370

[30] Wilmsen B. After the Deluge: A longitudinal study of resettlement at the Three Gorges Dam, China. World Development. 2016;84:41–54. doi: 10.1016/j.worlddev.2016.04.003

[31] Sun H, Gao G, Li Z. Research on the cooperative mechanism of government and enterprise for basin ecological compensation based on differential game. PLoS One. 2021;16(7):e0254411. doi: 10.1371/journal.pone.0254411 34298548 PMC8302252

[32] Chen J, Wang Q, Li Q. A Quantitative Assessment on Ecological Compensation Based on Water Resources Value Accounting: A Case Study of Water Source Area of the Middle Route of South-To-North Water Transfer Project in China. Front Environ Sci. 2022;10. doi: 10.3389/fenvs.2022.854150

[33] Wang Y, Zhu K, Xiong X, Yin J, Yan H, Zhang Y, et al. Assessment of the Ecological Compensation Standards for Cross-Basin Water Diversion Projects from the Perspective of Main Headwater and Receiver Areas. Int J Environ Res Public Health. 2022;20(1):717. doi: 10.3390/ijerph20010717 36613035 PMC9819099

[34] Shengwu Z, Juan H. Impact of interprovincial pairing assistance policies on sustainable agricultural development in Xinjiang of China. Sci Rep. 2025;15(1):8372. doi: 10.1038/s41598-025-92502-x 40069375 PMC11897137

[35] Yu X, Wan K, Chang T. Unintended consequences: China’s pairing assistance policy and carbon emissions in administrative border areas -evidence from China. J Environ Manage. 2025;375:124301. doi: 10.1016/j.jenvman.2025.124301 39862826

[36] IPCC. Climate change 2001: Impacts, adaptation and vulnerability. Cambridge: Cambridge University Press; 2001. https://www.ipcc.ch/report/ar3/wg2//

[37] Adger WN. Vulnerability. Global Environmental Change. 2006;16(3):268–81. doi: 10.1016/j.gloenvcha.2006.02.006

[38] Berkes F, Folke C. Linking social and ecological systems for resilience and sustainability. Environment and Development Economics. 1994; 4(2):237–42. doi: 10.1017/S1355770X99220165

[39] Metzger MJ, Rounsevell MDA, Acosta-Michlik L, Leemans R, Schröter D. The vulnerability of ecosystem services to land use change. Agriculture, Ecosystems & Environment. 2006;114(1):69–85. doi: 10.1016/j.agee.2005.11.025

[40] Polsky C, Neff R, Yarnal B. Building comparable global change vulnerability assessments: The vulnerability scoping diagram. Global Environmental Change. 2007;17(3–4):472–85. doi: 10.1016/j.gloenvcha.2007.01.005

[41] Pradyumna A, Sankam J. Tools and methods for assessing health vulnerability and adaptation to climate change: A scoping review. The Journal of Climate Change and Health. 2022;8:100153. doi: 10.1016/j.joclim.2022.100153

[42] Ramírez Eudave R, Ferreira TM. On the suitability of a unified GIS-BIM-HBIM framework for cataloguing and assessing vulnerability in Historic Urban Landscapes: a critical review. International Journal of Geographical Information Science. 2020;35(10):2047–77. doi: 10.1080/13658816.2020.1844208

[43] Howe PD, Yarnal B, Coletti A, Wood NJ. The Participatory Vulnerability Scoping Diagram: Deliberative Risk Ranking for Community Water Systems. Annals of the Association of American Geographers. 2013;103(2):343–52. doi: 10.1080/00045608.2013.754673

[44] Weis SWM, Agostini VN, Roth LM, Gilmer B, Schill SR, Knowles JE, et al. Assessing vulnerability: an integrated approach for mapping adaptive capacity, sensitivity, and exposure. Climatic Change. 2016;136(3–4):615–29. doi: 10.1007/s10584-016-1642-0

[45] Ducusin RJC, Espaldon MVO, Rebancos CM, De Guzman LEP. Vulnerability assessment of climate change impacts on a Globally Important Agricultural Heritage System (GIAHS) in the Philippines: the case of Batad Rice Terraces, Banaue, Ifugao, Philippines. Climatic Change. 2019;153(3):395–421. doi: 10.1007/s10584-019-02397-7

[46] Xu G, Kang M, Metzger M, Jiang Y. Vulnerability of the human-environment system in arid regions: the case of Xilingol grassland in northern China. Poliash J Environ Stud. 2014;23(5):13.

[47] Spielman SE, Tuccillo J, Folch DC, Schweikert A, Davies R, Wood N, et al. Evaluating social vulnerability indicators: criteria and their application to the Social Vulnerability Index. Nat Hazards. 2020;100(1):417–36. doi: 10.1007/s11069-019-03820-z

[48] Dilling L, Daly ME, Travis WR, Ray AJ, Wilhelmi OV. The role of adaptive capacity in incremental and transformative adaptation in three large U.S. Urban water systems. Global Environmental Change. 2023;79:102649. doi: 10.1016/j.gloenvcha.2023.102649

[49] Gariazzo C, Pelliccioni A, Bolignano A. A dynamic urban air pollution population exposure assessment study using model and population density data derived by mobile phone traffic. Atmospheric Environment. 2016;131:289–300. doi: 10.1016/j.atmosenv.2016.02.011

[50] Chen H, He W, Zhang S. Recent urbanization increases exposure to humid-heat extreme events over populated regions of China. Atmospheric and Oceanic Science Letters. 2024;17(2):100409. doi: 10.1016/j.aosl.2023.100409

[51] Gunasekera R, Ishizawa O, Aubrecht C, Blankespoor B, Murray S, Pomonis A, et al. Developing an adaptive global exposure model to support the generation of country disaster risk profiles. Earth-Science Reviews. 2015;150:594–608. doi: 10.1016/j.earscirev.2015.08.012

[52] Alam J. Impact of agriculture, industry and service sector’s value added in the gdp on co2 emissions of selected south asian countries. World Rev Bus Res. 2015;5(2):39–59.

[53] Sajjad RH, Rahaman MH, Masroor M, Sharma Y, Sharma A, et al. Vulnerability assessment of forest ecosystem based on exposure, sensitivity and adaptive capacity in the Valmiki Tiger Reserve, India: A geospatial analysis. Ecological Informatics. 2024;80:102494. doi: 10.1016/j.ecoinf.2024.102494

[54] Maja MM, Ayano SF. The Impact of Population Growth on Natural Resources and Farmers’ Capacity to Adapt to Climate Change in Low-Income Countries. Earth Syst Environ. 2021;5(2):271–83. doi: 10.1007/s41748-021-00209-6

[55] Hinkel J, Garcin M, Gussmann G, Amores A, Barbier C, Bisaro A, et al. Co-creating a coastal climate service to prioritise investments in erosion prevention and sea-level rise adaptation in the Maldives. Climate Services. 2023;31:100401. doi: 10.1016/j.cliser.2023.100401

[56] He C, Qiu W, Yu J. Climate Change Adaptation: A Study of Digital Financial Inclusion and Consumption Among Rural Residents in China. Front Environ Sci. 2022;10. doi: 10.3389/fenvs.2022.889869

[57] Azad MJ, Pritchard B. Financial capital as a shaper of households’ adaptive capabilities to flood risk in northern Bangladesh. Ecological Economics. 2022;195:107381. doi: 10.1016/j.ecolecon.2022.107381

[58] da Cunha C, Nikulkina I, Vanderlinden J-P, Shadrin V, Doloisio N, Salakhova D. Adaptive capacity for climate change: Local initiatives and federal planning. The case of Tiksi, Sakha Republic, Russia. Polar Science. 2022;31:100761. doi: 10.1016/j.polar.2021.100761

[59] Jiang B, Zheng H, Wang H, Zheng Y, Lin H, Wang Y. Study on the dust production characteristics of coal cutting at different drilling speeds of cutting head. Process Safety and Environmental Protection. 2025;193:1320–31. doi: 10.1016/j.psep.2024.11.128

[60] Xu A, Wu Y-H, Chen Z, Wu G, Wu Q, Ling F, et al. Towards the new era of wastewater treatment of China: Development history, current status, and future directions. Water Cycle. 2020;1:80–7. doi: 10.1016/j.watcyc.2020.06.004

[61] Wei L, Lin B, Zheng Z, Wu W, Zhou Y. Does fiscal expenditure promote green technological innovation in China? Evidence from Chinese cities. Environmental Impact Assessment Review. 2023;98:106945. doi: 10.1016/j.eiar.2022.106945

[62] Gong J, Jin T, Cao E, Wang S, Yan L. Is ecological vulnerability assessment based on the VSD model and AHP-Entropy method useful for loessial forest landscape protection and adaptative management? A case study of Ziwuling Mountain Region, China. Ecological Indicators. 2022;143:109379. doi: 10.1016/j.ecolind.2022.109379

[63] Su M, Wang Q, Li R, Wang L. Per capita renewable energy consumption in 116 countries: The effects of urbanization, industrialization, GDP, aging, and trade openness. Energy. 2022;254:124289. doi: 10.1016/j.energy.2022.124289

[64] Wang C, Qi W-K, Zhang S-J, Liu L-F, Peng Y-Z. Innovation for continuous aerobic granular sludge process in actual municipal sewage treatment: Self-circulating up-flow fluidized bed process. Water Res. 2024;260:121862. doi: 10.1016/j.watres.2024.121862 38908310

[65] Shi P, Li P, Li Z, Sun J, Wang D, Min Z. Effects of grass vegetation coverage and position on runoff and sediment yields on the slope of Loess Plateau, China. Agricultural Water Management. 2022;259:107231. doi: 10.1016/j.agwat.2021.107231

[66] Wu RMX, Zhang Z, Yan W, Fan J, Gou J, Liu B, et al. A comparative analysis of the principal component analysis and entropy weight methods to establish the indexing measurement. PLoS One. 2022;17(1):e0262261. doi: 10.1371/journal.pone.0262261 35085274 PMC8802816

[67] Jiang Y, Guan D, He X, Yin B, Zhou L, Sun L, et al. Quantification of the coupling relationship between ecological compensation and ecosystem services in the Yangtze River Economic Belt, China. Land Use Policy. 2022;114:105995. doi: 10.1016/j.landusepol.2022.105995

[68] Yu Y, Li J, Han L, Zhang S. Research on ecological compensation based on the supply and demand of ecosystem services in the Qinling-Daba Mountains. Ecological Indicators. 2023;154:110687. doi: 10.1016/j.ecolind.2023.110687

[69] Yang Y, Zhang Y, Yang H, Yang F. Horizontal ecological compensation as a tool for sustainable development of urban agglomerations: Exploration of the realization mechanism of Guanzhong Plain urban agglomeration in China. Environmental Science & Policy. 2022;137:301–13. doi: 10.1016/j.envsci.2022.09.004

[70] Ru Y, Zhang Y. A consideration of the enhancement of holistic ecological protection compensation mechanisms within Beijing ecological conservation zones. The people’s congress of Beijing. 2025;2:40–3

[71] Sheng J, Qiu W, Han X. China’s PES-like horizontal eco-compensation program: Combining market-oriented mechanisms and government interventions. Ecosystem Services. 2020;45:101164. doi: 10.1016/j.ecoser.2020.101164

[72] Metzger MJ, Leemans R, Schröter D. A multidisciplinary multi-scale framework for assessing vulnerabilities to global change. International Journal of Applied Earth Observation and Geoinformation. 2005;7(4):253–67. doi: 10.1016/j.jag.2005.06.011

